# Effect of the Finger Grip Relaxation Technique on Pain Levels Among Children Who Underwent Abdominal Surgery

**DOI:** 10.7759/cureus.77741

**Published:** 2025-01-20

**Authors:** Rohini P Dani, Dhanraj Babu

**Affiliations:** 1 Nursing, Bharati Vidyapeeth Deemed to be University, Pune, IND; 2 Child Health Nursing, Bharati Vidyapeeth Deemed to be University, Pune, IND

**Keywords:** abdominal surgery, effect, finger grip, pain, relaxation technique

## Abstract

Background: Children have a fundamental right to be free from pain, which can be managed through pharmaceutical and non-pharmacologic techniques, often most effective when combined. Pain is closely linked to tension, anxiety, and worry, but non-pharmacologic methods like guided visualization and cutaneous stimulation can reduce anxiety and alter pain perception. The finger grip relaxation technique, involving finger grasping, deep breathing, and muscle relaxation, helps relieve tension and improve pain tolerance. This method is suitable for use at home and in hospitals to reduce postoperative pain. The objectives are to assess pain levels after intervention in control and experimental groups and to compare postintervention pain levels between the two groups.

Methodology: This study employed a quantitative research approach and utilized a quasi-experimental design, specifically a pretest-posttest design with a control group. The sample of the study consists of 60 children who had undergone abdominal surgery in the selected hospitals from the day of surgery after recovery from anesthesia. Participants were assigned into two groups, and the experimental group received the intervention finger grip relaxation technique for 30 minutes twice a day for four days. Based on the study objectives, demographic data and the numerical pain rating scale were used for the collection of data.

Result: In the experimental group, 63% (19) of the samples had mild pain and 37% (11) had moderate pain, whereas in the control group, 70% (21) of the samples had moderate pain and 30% (9) had severe pain. The posttest experimental group had a mean of 3.1 with a standard deviation (SD) of 0.7119, the control group had a mean of 5.96 with an SD of 1.3257, the t-value was 10.4343, and the p-value was 0.00001, which is less than 0.05. It shows that the finger grip relaxation technique is effective in reducing surgical incisional pain among children who had undergone abdominal surgery.

Conclusion: The study concluded that children who had abdominal surgery experienced less pain in the experimental group compared to the control group. This shows that the finger grip relaxation technique was effective in reducing surgical pain. Before using the technique, most children in both groups had severe pain. However, after the technique was applied, the experimental group showed a significant reduction in pain levels compared to the control group.

## Introduction

Pain is defined by the International Association for the Study of Pain (IASP) as "an unpleasant sensory and emotional experience associated with actual or potential tissue damage or described in terms of such damage" [1]. Postoperative pain is a significant issue for most surgical patients, often leading to physical and emotional distress. Among various procedures, abdominal surgeries are noted to cause some of the most intense pain due to the region’s proximity to the diaphragm and the complex network of nerves in the area. Poorly managed postoperative pain can result in complications such as breathing difficulties, sleep disturbances, appetite loss, prolonged hospital stays, patient dissatisfaction, and increased healthcare costs [2].

Effective pain management is vital for improving recovery outcomes. Pain perception can lead to fatigue, reducing patients’ ability to engage in self-care or recovery. Children, in particular, are vulnerable to untreated pain, which can negatively impact their emotional and psychological health. Unlike adults, children may struggle to communicate their pain levels, necessitating individualized assessment and care [3].

Pain management strategies are typically divided into pharmaceutical and non-pharmaceutical approaches, with the best outcomes often achieved through a combination of both. Non-pharmaceutical techniques, such as guided visualization, cutaneous stimulation, and relaxation exercises, are particularly beneficial in addressing the psychological aspects of pain [4]. The finger grip relaxation technique, for instance, involves holding specific fingers to reduce stress and tension, stimulating energy channels known as meridians. This practice is thought to produce endorphins-natural painkillers in the body-while also promoting emotional stability and physical relaxation [5].

The finger grip relaxation method incorporates elements like deep breathing, muscle relaxation, and targeted finger grasping. These practices can be applied both at home and in hospital settings to alleviate pain and enhance recovery. By focusing on relaxation and energy flow, this technique helps patients manage stress, thereby increasing their tolerance for pain and improving their overall well-being [6].

Pain is a subjective experience that requires careful assessment and management. Effective strategies, particularly for postoperative care, involve a blend of pharmaceutical and non-pharmaceutical methods. Non-pharmacological interventions, such as relaxation techniques, can significantly enhance pain management outcomes, benefiting both physical recovery and emotional well-being [7,8].

Pain is a common and significant issue following abdominal surgeries in children. Postoperative pain can range from mild to severe and, if poorly managed, may lead to complications like delayed recovery, increased anxiety, and longer hospital stays [9]. Children often experience challenges in expressing their pain, making assessment and management more complex.

This study addresses the significant challenge of postoperative pain, particularly in abdominal surgeries, where pain intensity is high. By integrating non-pharmacological methods like the finger grip relaxation technique, such methods provide psychological and emotional benefits, alleviating anxiety and promoting well-being. Therefore, the researchers selected the finger grip relaxation technique since it is practical, cost-effective, and easy to implement across diverse settings, making it widely accessible.

## Materials and methods

The present study aimed to evaluate the effectiveness of the finger grip relaxation technique in reducing pain levels among children who underwent abdominal surgery. A quantitative research approach was adopted to assess the existing level of pain, allowing for objective measurement. The research design was quasi-experimental, utilizing a pretest-posttest control group design. The independent variable was the finger grip relaxation technique, and the dependent variable was the level of pain experienced by the children [9]. The study was conducted at selected pediatric surgical hospitals. The target population included children aged 6-14 years admitted for abdominal surgery. The sample size was determined using power analysis to ensure adequate statistical power for the study. The calculated sample size was 60 children, with 30 participants in the control group and 30 in the experimental group. Non-probability purposive sampling was used to select participants. The inclusion criteria were children aged 6-14 years who had undergone abdominal surgery and were recovering from anesthesia. The exclusion criteria included children whose parents did not provide written consent and those with cognitive impairments. The ethical approval was obtained from the Institutional Ethical Committee (IEC) IECBVDUCONSANGLI-EC/NEW/INST/2024/MH/0414. The research was approved by the IEC on April 8, 2024. Formal permission was taken from the concerned authorities of selected hospitals. Those who were willing to participate were included, and informed written consent was taken from the parents of participants after explaining the purpose of the study.

Study participants and intervention

Non-probability purposive sampling was used to select participants, who were assigned to either the control or experimental group. A preassessment was conducted before the intervention, during which the investigator performed the finger grip relaxation technique on the experimental group for 30 minutes, twice daily, over four days. A postassessment was conducted on the fourth day. The study was completed over one month and one week, spanning July and August 2024. The inclusion criteria included children aged 6-14 years who underwent abdominal surgery, who were assessed postrecovery from anesthesia, and who provided consent. The exclusion criteria included children with cognitive impairments or those whose parents did not consent in writing.

The finger grip relaxation technique is a simple, non-invasive method used to relieve pain in children after abdominal surgery. It begins by ensuring the child is comfortable, with their hands resting on a soft cushion, shoulders relaxed, and breathing steady. The process includes two to three minutes of deep breathing, followed by gently gripping each finger of the opposite hand, applying light pressure from the thumb to the pinky, squeezing each for five seconds, and then releasing slowly with a deep breath. This is repeated on the other hand. The technique promotes relaxation, reduces pain by stimulating pressure points, and calms the nervous system. It is an effective distraction tool and requires no special equipment. The control group received regular hospital treatment.

Statistical analysis

The data analysis focused on demographic variables, which were computed using descriptive and inferential statistics. IBM SPSS Statistics for Windows, Version 28.0 (IBM Corp., Armonk, NY, US), was used for the analysis. Frequency and percentage distributions were applied to evaluate the demographic variables.

The mean and standard deviation (SD) were used to assess the postintervention pain levels of children who had undergone abdominal surgery in selected hospitals. An unpaired t-test was used to evaluate the effectiveness of the finger grip relaxation technique in reducing pain levels among these children.

## Results

Table 1 shows the maximum number of children from the age group between six and eight years in both groups. In terms of gender, 43% were boys in the control group and 57% in the experimental group; 57% were girls in the control group and 43% in the experimental group. In both groups, 100% of children had no experience with surgery.

**Table 1:** Baseline demographic data. Frequency and percentage distribution of demographic variables

Sr. No.	Demographic variables		Experimental group		Control group	
			F	%	F	%
1	Age (in years)	6-8	13	43	12	40
		9-11	6	20	11	37
		12-14	11	37	7	23
2	Gender	Male	17	57	13	43
		Female	13	43	17	57
3	Previous experience with surgery	Yes	0	0	0	0
		No	30	100	30	100

Table 2 shows that, in the pretest, 77% of children had severe pain and 23% had moderate pain in the experimental group, whereas in the control group, 70% of children had severe pain and 30% had moderate pain.

**Table 2:** Frequency and percentage distribution of the pretest surgical level of pain

Pretest	Experimental group		Control group	
	Frequency (f)	Percentage (%)	Frequency (f)	Percentage (%)
Mild pain (1-3)	0	0	0	0
Moderate pain (4-6)	7	23	9	30
Severe pain (7-10)	23	77	21	70

Table 3 shows that 63% of the samples had mild pain and 37% had moderate pain in the experimental group, whereas in the control group, 70% of the samples had moderate pain and 30% had severe pain. Hence, it is concluded that the majority of the samples had mild pain in the experimental group after the intervention of finger grip relaxation.

**Table 3:** Frequency and percentage distribution of the posttest level of surgical incisional pain

Posttest	Experimental group		Control group	
	Frequency (f)	Percentage (%)	Frequency (f)	Percentage (%)
Mild pain (1-3)	19	63	0	0
Moderate pain (4-6)	11	37	21	70
Severe pain (7-10)	0	0	9	30

Table 4 shows a difference in surgical incisional pain between the groups. The experimental group had an average pain score of 3.1 (SD = 0.7119), while the control group had a score of 5.96 (SD = 1.3257). The t-value was 10.4343, and the p-value was 0.00001, which is less than 0.05. This suggests that the finger grip relaxation technique is effective in reducing surgical incisional pain in children after abdominal surgery.

**Table 4:** Comparison between posttest scores in the experimental and control groups SD: standard deviation

Posttest	Mean	SD	t-value	p-value	Significance
Experimental group	3.1	0.7119	10.4343	0.00001	Significant
Control group	5.96	1.3257			

## Discussion

The study was conducted to assess the effectiveness of the finger grip relaxation technique on the level of pain among children who had undergone abdominal surgery in selected surgical hospitals.

Among the total sample, in the experimental group, 43% belongs to the age group between six and eight years, 37% between 12 and 14 years, and 20% between nine and 11 years, whereas in the control group, 40% belongs to the age group between six and eight years, 37% between nine and 11 years, and 23% between 12 and 14 years. Regarding the gender of the children, in the experimental group, 57% are boys and 43% are girls, whereas in the control group, 57% are girls and 43% are boys. Among the 60 participants, all of the children, i.e., 100% in the experimental group and 100% in the control group, had no previous experience with surgery. The control group had severe and moderate pain as compared to the experimental group. Findings are related to the effectiveness of the finger grip relaxation technique on the level of pain in children who had undergone abdominal surgery. There is a statistically significant change in the level of pain.

In the pretest, 77% of children had severe pain and 23% had moderate pain in the experimental group, whereas in the control group, 70% of children had severe pain and 30% had moderate pain. Hence, it is concluded that the majority of children who had undergone abdominal surgery faced severe pain in both the experimental and control groups.

In the posttest, 63% of the samples had mild pain and 37% had moderate pain in the experimental group, whereas in the control group, 70% of the samples had moderate pain and 30% had severe pain. Hence, it is concluded that the majority of the samples had mild pain in the experimental group after the intervention of the finger grip relaxation technique. The samples had severe pain in the control group. It concludes that the level of pain among children who had undergone abdominal surgery decreased in the experimental group as compared to the control group. A similar study by Calisanie and Ratnasari assessed the effectiveness of the finger grip relaxation technique in reducing pain intensity in postappendectomy patients. The influence of finger relaxation techniques on pain reduction in patients postappendectomy was evaluated in this study using a literature review methodology. Patients undergoing appendectomy surgery complained of severe pain before being taught the finger grip relaxation technique. After using the technique, patients' discomfort effectively decreased. After analyzing the data with the paired t-test, p < 0.05 was determined to be the significant value. The average result was 4.80 before the intervention and 3.87 after it, according to the data. The p-value for the two-variable results was 0.000. It illustrates how pain intensity varies in postappendectomy patients both before and after finger grip relaxation treatments are applied. Both in the hospital and at home, finger grip relaxation techniques can be used to reduce discomfort in patients recovering from postappendectomy [10].

Additionally, a study by Kumar et al. evaluated the effectiveness of finger grip relaxation techniques in postsurgical pain management for pediatric patients. Their results indicated a significant decrease in pain intensity and anxiety in children who used the finger grip technique compared to those who did not. This study highlighted the technique's potential as a non-invasive and effective tool for pain management in pediatric care [11].

The recommendation of the studies is that a similar study can be conducted on a larger sample size to enhance the generalizability of the findings. Comparative studies can be done to evaluate the effectiveness of different non-pharmacological pain relief techniques. This study was limited to children who underwent abdominal surgery in selected hospitals only.

## Conclusions

The study evaluated the effectiveness of the finger grip relaxation technique in reducing postoperative pain in children who underwent abdominal surgery. Results revealed a significant reduction in pain levels in the experimental group compared to the control group. Before the intervention, most children in both groups reported severe pain. After the technique was applied, 63% of children in the experimental group experienced mild pain and 37% reported moderate pain, whereas the control group continued to report higher pain levels. These findings highlight the technique's effectiveness as a non-pharmacologic pain management tool, particularly when used alongside pharmacologic interventions.

The study underscores the importance of integrating both pharmacologic and non-pharmacologic methods, such as relaxation techniques, into routine postoperative care for children to achieve better pain relief outcomes. Further research with a larger sample size and comparisons to other multimodal pain management approaches are recommended to enhance the understanding and application of the finger grip relaxation technique in pediatric care.
